# Single-Cell RNA-Sequencing: Opening New Horizons for Breast Cancer Research

**DOI:** 10.3390/ijms25179482

**Published:** 2024-08-31

**Authors:** Lingyan Xiang, Jie Rao, Jingping Yuan, Ting Xie, Honglin Yan

**Affiliations:** Department of Pathology, Renmin Hospital of Wuhan University, Wuhan 430060, China; 2023283020213@whu.edu.cn (L.X.); rm002228@whu.edu.cn (J.R.); yuanjingping@whu.edu.cn (J.Y.); 2023283020214@whu.edu.cn (T.X.)

**Keywords:** single-cell RNA-sequencing, breast cancer, heterogeneity, tumor microenvironment, therapy, drug resistance

## Abstract

Breast cancer is the most prevalent malignant tumor among women with high heterogeneity. Traditional techniques frequently struggle to comprehensively capture the intricacy and variety of cellular states and interactions within breast cancer. As global precision medicine rapidly advances, single-cell RNA sequencing (scRNA-seq) has become a highly effective technique, revolutionizing breast cancer research by offering unprecedented insights into the cellular heterogeneity and complexity of breast cancer. This cutting-edge technology facilitates the analysis of gene expression profiles at the single-cell level, uncovering diverse cell types and states within the tumor microenvironment. By dissecting the cellular composition and transcriptional signatures of breast cancer cells, scRNA-seq provides new perspectives for understanding the mechanisms behind tumor therapy, drug resistance and metastasis in breast cancer. In this review, we summarized the working principle and workflow of scRNA-seq and emphasized the major applications and discoveries of scRNA-seq in breast cancer research, highlighting its impact on our comprehension of breast cancer biology and its potential for guiding personalized treatment strategies.

## 1. Introduction

Breast cancer is characterized by the aggressive proliferation of ducts and mammary epithelial cells throughout various layers of breast tissue. The disease progression typically starts with premalignant atypical ductal hyperplasia (ADH), followed by the development of ductal carcinoma in situ (DCIS) and ultimately culminating in the formation of malignant invasive ductal carcinoma (IDC) [1]. It ranks highest in incidence among malignant tumors affecting women [2]. Breast cancer has possession of distinct molecular subtypes, namely luminal A, luminal B, human epidermal growth factor receptor-2 positive (HER2+) and triple-negative breast cancer (TNBC). Patients with different subtypes of breast cancer have varying clinical prognoses, thus necessitating diverse therapeutic approaches tailored to specific molecular subtypes. However, even among different breast cancer patients with the same molecular typing, there still exist significant differences in their clinical outcomes or prognoses. Many problems that appear in the treatment process, such as drug resistance and immune escape, bring great challenges to the treatment of breast cancer. Notably, multiple omics studies have revealed the presence of both heterogeneity within and between tumors in breast cancer is the primary reason for relapse or drug resistance [3,4,5]. Identifying the specific heterogeneity characteristics of breast cancer brings benefits such as individualized treatment, accurate prognosis, and the discovery of therapeutic targets, and has clinical significance in terms of enhancing the understanding of the disease mechanism, improving the diagnosis, and allowing for better monitoring and follow-up [6].

The variation in the tumor microenvironment (TME) is an important factor contributing to tumor heterogeneity. Cancer cells profoundly shape the heterogeneous population of tissue-resident and tumor-infiltrating cells that make up the stromal compartment of the TME [7]. The TME encompasses cancer cells, all benign cells (including stromal cells, immune cells, vascular endothelial cells) and components within the tumor, as well as the molecules that they produce and release, all of which interact with the tumor cells and influence their behavior and properties [8]. This interaction can contribute to the identified variability and play critical roles in the occurrence, growth, metastasis, and promotion of tumor cell growth and migration in breast cancer [9]. Understanding this complexity and heterogeneity of TME in breast cancer is crucial for developing effective therapeutic approaches.

Previous research on the composition of the TME (such as flow cytometry, multiplex immunohistochemistry/immunofluorescence techniques, etc.) is merely confined to the analysis of a few markers, making it hard to fully reflect the complex diversity and heterogeneity of the TME. For instance, in the case of flow cytometry (FCM), the spectral overlap between different fluorochromes can pose challenges in properly assigning fluorescence signals, and limit the number of detected markers [10,11]. Similarly, while multiplex immunohistochemistry/immunofluorescence techniques (mIHC/IF) enable the visualization of multiple biomarkers on a single slide, they still fall short in terms of providing a comprehensive understanding at the genomic level [12]. The emergence of bulk RNA sequencing enables researchers to sequence the RNAs in an entire tissue sample consisting of a mixture of multiple cells with higher throughput and efficiency, thus revealing the full picture of gene expression and providing an important tool for the study of the TME [13]. However, the gene expression levels obtained from this technique represent the average expression across all the cells in the sample, making it unsuitable for characterizing cell heterogeneity [14]. With the rapid development of global precision medicine, single-cell RNA sequencing (scRNA-seq) has emerged as a powerful tool, revolutionizing breast cancer research by offering unprecedented insights into the cellular heterogeneity and complexity of breast cancer. This cutting-edge technology facilitates the profiling of gene expression at the single-cell level, uncovering diverse cell types and states within the TME [15].

In this review, we summarized the working principle and workflow of scRNA-seq and emphasized the major applications and discoveries of scRNA-seq in breast cancer research, such as exploring the heterogeneity and TME of breast cancer and its progress in treatment research and exploring the mechanism of drug resistance and distant metastasis after treatment. This review has deepened the understanding of scRNA-seq in breast cancer biology and its potential to guide personalized treatment strategies.

## 2. The Functional Principle and Workflow of scRNA-Seq

**Figure 1:**
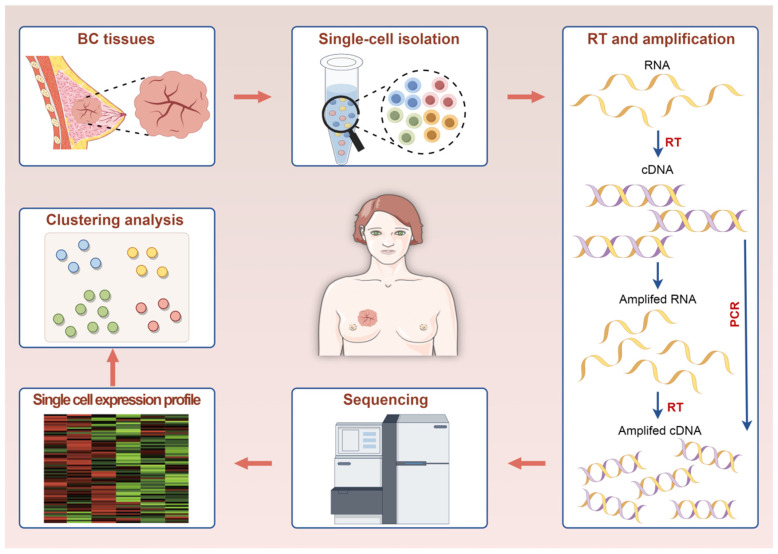
Workflow of a typical single-cell RNA sequencing experiment.

Eberwine [16] and his colleagues were the pioneers of single-cell transcriptome sequencing. They utilized linear amplification through in vitro transcription to amplify the complementary DNAs (cDNAs) of a single cell. ScRNA-seq captures transcriptional heterogeneity at the single-nucleotide level within individual cells and detects differences in gene expression across diverse cell types [17]. This sequencing technique requires the extraction of individual cells from the specimens at the beginning [18,19]. The main processes involved in scRNA-seq technology can be summarized as follows: single-cell isolation, reverse transcription (RT), amplification, library preparation, sequencing, and data analysis. Figure 1 introduces the workflow of scRNA-seq.

### 2.1. Single-Cell Isolation

To analyze mRNA in each cell individually, the initial step is to isolate a single cell from the samples [20]. At present, there are four main methods for isolating single cells.

Micromanipulation: Micromanipulation involves the use of a micropipette or gently sucking single cells through an oral straw under a microscope, which can ensure that each sample is indeed a single cell [21,22]. However, it should be noted that micromanipulation has limitations in terms of its ability to pick up only a limited number of cells, and it requires a high level of proficiency in experimental operation skills [22]. These shortcomings have led to a decrease in the utilization of micromanipulation in current research practices.

Laser capture microdissection (LCM): LCM employs laser technology for cutting and capturing single cells from solid samples [18]. This technique is particularly useful for samples or biopsies that cannot be easily dissociated into single-cell suspensions [23]. Infrared (IR) and ultraviolet (UV) are two commonly utilized technologies for LCM. IR LCM uses a thermolabile polymer that is directly applied directly onto the tissue, and this polymer melts when an IR laser pulse is applied, forming a composite of polymer and cells that can be separated. UV LCM involves cutting the region of interest (ROI) on a polyethylene terephthalate (PET) membrane slide using a UV laser, with isolated cells captured in a tube below the tissue slide. Both technologies allow for precise cell isolation from tissue sections [24]. LCM enables the isolation of individual cells or specific ROI under visual guidance, but it has certain limitations, such as high cost, specialized personnel requirements, and potential tissue quality compromise due to the absence of coverslips [25].

Microfluidics: Microfluidics is currently the most widely used method for acquiring individual cells, capturing them by regulating fluid movement within a microfluidic device [26]. It is regarded as a miniaturized “chip lab”, which streamlines labor-intensive operations into microdevices and enables precise interrogation of individual cells. In single-cell omics research, microfluidic chips enhance analysis sensitivity and accuracy through reduced reaction volumes, isolated chambers to prevent contamination, and sequential manipulations on a single chip, ultimately facilitating high-throughput single-cell analysis [27]. Two popular microfluidic systems are the 10× Genomics Chromium and FluidigmC1 systems, which have been demonstrated to provide multiple advantages over platforms that utilize droplets for capturing single cells [28,29]. The advantages, such as enhancing sensitivity, precision, and processing capacity for analysis, promote the development of microfluidic-assisted single-cell transcriptome analysis to focus on achieving high throughput, reducing costs, and simplifying operation [27]. These advantages have contributed to the increasing popularity of microfluidics.

Fluorescence-activated cell sorting (FACS): FACS is a specialized form of FCM, and its working principle is that cells labeled with specific fluorescent probes emit fluorescence and scatter light when passing through a laser beam [30]. The main advantages of FACS are the high throughput capabilities and fast separation speed. However, it is important to note that while FACS is suitable for obtaining individual cells from sections of tissue or cultured cell aggregates, it is not optimal for capturing rare cells like tumor cells circulating in the bloodstream or tumor cells spread in the bone marrow [31].

Generally, these methods have their own advantages. Micromanipulation can ensure each sample is indeed a single cell. LCM allows for the isolation of individual cells or specific regions of interest. The microfluidic approach has high throughput capabilities and lower analysis costs. FACS has high throughput capabilities and fast separation speed. The selection of the optimal method depends on specific criteria for capturing single cells.

### 2.2. Reverse Transcription, Amplification, and Sequencing

Obtaining adequate cDNA from limited RNA within a single cell is crucial for transcriptomic analyses at the single-cell level. The efficiency of RT and amplification significantly influences the precision and detection capability of scRNA-seq. Different RT reactions and cDNA amplification strategies cause different operation protocols of scRNA-seq. After the amplification, the library preparation will be performed on the amplified cDNA, and then the resulting samples will be loaded onto the computer and sequenced. There are three existing principles for single-cell transcriptome library construction and sequencing: polymerase chain reaction (PCR) after polyA tailing, template-switching-based PCR, and in vitro transcriptional (IVT) amplification. Under these three principles, many different sequencing methods have been subdivided. Details are shown in Table 1.

#### 2.2.1. PCR after polyA Tailing

The amplification method of PCR after polyA tailing is by employing polyA tailing, through which the mRNA with polyA tail binds to polyT primers with a unique molecular identifier (UMI) and a tag specific to individual cells. There are five main sequencing methods based on this approach, each with its own unique process and technology. The earliest technology is the Tang method, developed by Tang et al. [21]. This method involves directly cleaving single cells and converting the resulting mRNA into cDNA through RT using oligonucleotide-dT primers containing the anchor sequence UP1. To ensure the production of complete-length cDNA, terminal deoxynucleotide transferase is employed to append a polyadenylate tail to the 3′ end of the initial cDNA strand, thereby extending its sequence. The second strand of cDNA is then synthesized using an oligonucleotide-dT primer of another anchoring sequence UP2, followed by PCR amplification using the UP1 and UP2 primers. Similar to the Tang method, Quartz-seq [32] also involves the direct conversion of single-cell RNAs into cDNA through RT with an RT primer. The first-strand cDNAs are then generated from the target RNAs, and primer digestion using exonuclease I is carried out to prevent the formation of unwanted side products. A poly-A tail is then appended to the 3′ ends of the first-strand cDNAs, followed by second-strand synthesis and PCR amplification to ensure sufficient DNA quantity. Based on Quartz-seq, researchers have developed a new method. Quartz-seq2 [33] employs a cell sorter to isolate viable single cells into a 384-well PCR plate containing lysis solution, excluding non-viable cells. Each well contains a lysis solution and an RT primer with a cell barcode, UMI, and a -dT sequence. RNA from each cell is converted to cDNA using these RT primers. The first-strand cDNA is then extended with a polyA tail, followed by second-strand cDNA synthesis and PCR amplification to pool cDNA from multiple single cells into a combined mixture. The other technique is the SUPeR-seq [34,35] which begins with the lysis of a single cell to release its RNA content, followed by RT to use random primers containing a fixed anchor sequence to synthesize first-strand cDNAs from the RNA molecules. PolyA tails are added to the 3′ ends of the first-strand cDNAs, followed by second-strand cDNA synthesis and PCR amplification to ensure a comprehensive representation of the cDNA population for downstream analysis. The last way is MATQ-seq [36]. It involves first-strand cDNA synthesis using a combination of dT and random primers via multiple annealing, followed by enzymatic digestion to eliminate residual primers and RNA molecules. The 3′ ends of the first-strand cDNAs are then modified via polyC tailing, and second-strand synthesis and PCR amplification are performed before library construction and sequencing evaluation.

#### 2.2.2. Template-Switching-Based PCR

The other approach for amplification is by using a template-switching (TS) mechanism. There are numerous sequencing techniques based on the template-switching PCR amplification method. The most typical one is SMART-seq, which employs M-MLV reverse transcriptase for the RT process, resulting in the addition of cytosines to the 3′ end, enabling complete template conversion at the 5′ end. M-MLV reverse transcriptase prefers full-length cDNAs as substrates for its terminal transferase activity, enabling the coupling of RT and TS in a single reaction. Moreover, the SMART-seq protocol uses special primers to ensure consistent PCR amplification efficiency for cDNA synthesis. This template-switching mechanism allows for the synthesis of full-length cDNA from RNA, capturing the entire transcript length and reducing 3′ coverage biases from incomplete RT [26]. This is crucial for understanding alternative splicing, polyadenylation sites, and other regulatory elements that can vary along the length of a transcript [37,38,39,40]. SMART-seq2 is an improvement that enhances cDNA yield and sensitivity by introducing a locked nucleic acid modification. SMART-seq3 further enhances sensitivity and employs tagmentation with Tn5 to produce 5′ UMI-tagged and internal fragments [37,38,39,40]. SMART-seq introduces known sequences flanking both ends of each transcript through TS and oligonucleotide-dT priming, enabling PCR amplification using a solitary primer set. Conversely, FLASH-seq combines these steps in a single RT-PCR reaction. FLASH-seq adopts a two-step amplification approach, starting with the RT to synthesize full-length cDNA, followed by PCR amplification. Similar to all SMART-seq methods, FLASH-seq necessitates the further preparation of cDNA for sequencing, which involves the use of a hyperactive Tn5 transposase [41]. It should be noted that compared with SMART-seq3, FLASH-seq has higher sensitivity and a shorter operation time. The FLASH-seq protocol can also utilize UMI for molecular counting; moreover, it shows reduced strand-invasion artifacts, typical full-length genome coverage, and improved intercellular correlation. Under the same number of PCR cycles, FLASH-seq generates eight times more cDNA than SMART-seq2 and SMART-seq3 [41]. Therefore, it is a more efficient approach for preserving full-length transcriptome information and eliminating bias. STRT-seq is another method involving cell isolation, cDNA synthesis with barcoding, and subsequent amplification and sequencing. The method has been refined to incorporate UMIs for precise transcript quantification and can be performed on the FluidigmC1 platform. Another variation, STRT-seq-2i, utilizes a high-throughput microwell array platform for efficient multiplexing and single-read sequencing libraries [42,43]. SCRB-seq, in addition, utilizes fluorescence-activated cell sorting to sort cells into 96/384-well plates, incorporating cell barcodes and UMIs during RT for unbiased transcript quantification [44]. Drop-Seq is a method that uses tiny droplets to analyze mRNA expression in individual cells. It involves encapsulating cells with barcoded microparticles in droplets, lysing the cells, capturing the mRNA on the microparticles, and sequencing the transcripts. This platform allows for high-throughput profiling of thousands of cells while retaining information on the cell of origin for each transcript [45,46].

#### 2.2.3. In Vitro Transcription (IVT)

Both of the above two scRNA-seq methods share a major limitation: they are exclusively DNA-based [47]. Currently, the direct sequencing of RNA from single cells is not feasible, necessitating the conversion of RNA to cDNA and subsequent amplification, either through PCR or linear amplification [48]. The goal of amplification is to amplify DNA without bias, with completeness and uniformity being factors. During cDNA amplification, template-switching is frequently coupled with PCR, which is speedy and widespread. However, PCR has the disadvantage of inducing exponential amplification, resulting in an over-representation of highly expressed transcripts in the final library. Furthermore, due to variations in efficiency across different transcript species, PCR amplification introduces quantification biases, leading to the loss of original transcript abundance information and the accumulation of non-specific transcript fragments. There is a novel amplification technique that overcomes these limitations, namely linear amplification through IVT. This method initiates with an RT and cDNA synthesis using an oligonucleotide-dT primer that integrates a T7 promoter sequence. Subsequently, the T7 polymerase attaches to the T7 promoter and amplifies the RNA, followed by another round of RT. However, it has lower sensitivity for transcripts expressed at low levels. IVT requires more effort compared to PCR because it involves an extra round of reverse transcription to amplify the RNA. This extra step may lead to premature termination during transcription, resulting in the accumulation of RNA fragments biased towards the 3′ end (strong 3′-bias). This bias might limit the ability to fully capture the transcriptome landscape of the sample [49]. Despite its limitations, IVT offers the advantage of linear amplification, unlike the exponential amplification observed in PCR. For example, even if the sensitivity of this scheme to low-expression transcripts is relatively low, it does not exponentially deplete sequences that are hard to process, like PCR. Therefore, this linear amplification method can reduce the bias caused by PCR index amplification, improve accuracy, and better preserve the relative abundances of different RNA species. In some cases, linear amplification can accurately reflect RNA molecule proportions and is important for measuring expression level differences between samples. A recent advancement in genomic amplification, called multiple annealing and looping-based amplification cycles (MALBAC), has introduced quasi-linear preamplification. This technique aims to minimize the bias that is typically associated with nonlinear amplification methods [50]. The process combines an initial amplification stage using an enzyme capable of strand displacement with a subsequent phase of amplification through PCR. In this approach, DNA is initially amplified by an enzyme that displaces strands, producing fragments with matching ends. These matching ends allow the fragments to form loops, preventing them from being used as templates in later cycles, thus achieving a nearly linear amplification. After five rounds of this initial amplification, the material is then amplified exponentially through PCR [51]. This approach aims to reduce bias and improve the representation of the original sample in the amplified material.

There are five sequencing methods based on IVT. CEL-Seq and its improved version, CEL-Seq2, are techniques developed for sequencing RNA from individual cells. CEL-Seq uses a primer that binds to the polyA tail of RNA molecules, incorporating a unique barcode specific to each cell. This method focuses on sequencing the 3′ ends of the RNA transcripts. This early barcoding allows for early sample pooling, reducing hands-on time by creating a single library. With CEL-Seq2, the UMIs approach is implemented to further reduce amplification biases. CEL-seq2 builds upon CEL-seq by introducing UMIs and improving the efficiency of RT while shortening the length of barcode, adaptor, and T7 promoter primers. These techniques allow for the effective linear amplification of RNA from individual cells, facilitating their analysis through sequencing [52,53,54]. The MARS-seq framework enables high-throughput in vivo sampling of thousands of cells through multiplexed RNA sequencing while effectively managing amplification biases and labeling errors. This method uses FACS sorting to isolate individual cells and place them into 384-well plates. Subsequent automated processing, primarily conducted on pooled and labeled material, greatly enhances throughput and reproducibility [55]. MARS-seq2.0 is an enhanced version of the previously developed MARS-seq method, based on sequencing more than 1 million cells. This approach allows for the identification of distinct cell types across various tissues, diseases, experimental models, and species. The data analysis includes evaluating quality and detecting errors, providing essential statistics on the diversity of the library, the distribution of background noise, and the completeness of sequencing. The integration of FACS and scRNA-seq facilitates intuitive strategies for depleting or enriching cell populations, which is crucial for the efficient sampling of complex tissues [56]. In Drops is similar to Drop-seq, as it also utilizes droplet microfluidic technology to encapsulate cells, lysate, RT reagents, and hydrogel particles within a droplet. It enables the automatic completion of cell lysis and cDNA synthesis within the droplet. In contrast to Drop-seq, the gel beads used in In Drops incorporate the T7 RNA polymerase promoter [57].

### 2.3. Data Analysis

Analysis tools for scRNA-seq data are available in various programming languages, with R and Python being the most prominent choices [58]. While cross-environment support is increasing, the selection of a programming language often determines the available analysis tools. Established platforms like Seurat, Scater, or Scanpy offer comprehensive environments for building pipelines and come equipped with extensive analysis toolkits [59,60,61]. However, these platforms tend to be limited to tools developed specifically for their respective programming languages due to practical constraints [20].

**Figure 2:**
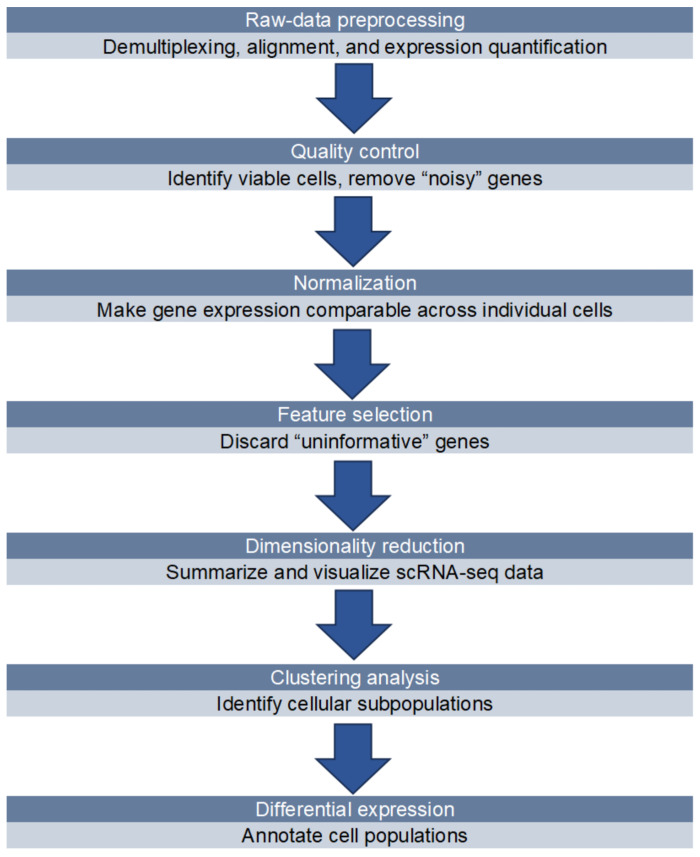
ScRNA-seq data basic analysis framework.

The standard data analysis workflow for scRNA-seq can be broken down into three main stages: processing and quality control of raw data, basic analysis applicable to most scRNA-seq datasets (Figure 2), and advanced analysis tailored to specific research contexts. The fundamental analysis steps include data normalization and merging, selecting key features, reducing dimensions, grouping cells into clusters, identifying cell types, and finding marker genes. Advanced analysis tasks involve tracing developmental pathways, analyzing cell–cell communication (CCC), predicting regulatory networks and transcription factor activities, and estimating metabolic pathways [62,63]. A lot of important information can be learned by analyzing scRNA-seq data. For example, through a series of steps such as dimensionality reduction clustering and cell annotation, the cell composition of the sequencing sample can be obtained, and its heterogeneity can be analyzed. Beyond capturing the differences between individual cells, scRNA-seq can also reveal the dynamic changes in transcriptomes that reflect developmental progressions or shifts in cell states. Techniques like developmental pathway analysis and pseudo-time estimation are essential for uncovering the molecular features and regulatory processes involved in cell differentiation or activation [64]. In addition, the analysis of CCC can observe the communication between cells. Intercellular communication plays a crucial role in numerous biological processes of multicellular organisms, including tissue homeostasis, development, and immune responses. Having a comprehensive understanding of how cells communicate can help regulate critical biological pathways and potentially improve cell-based therapies [65]. In conclusion, single-cell data analysis can provide considerable biological information, which is beneficial for disease research.

## 3. Application of scRNA-Seq in Breast Cancer

**Figure 3:**
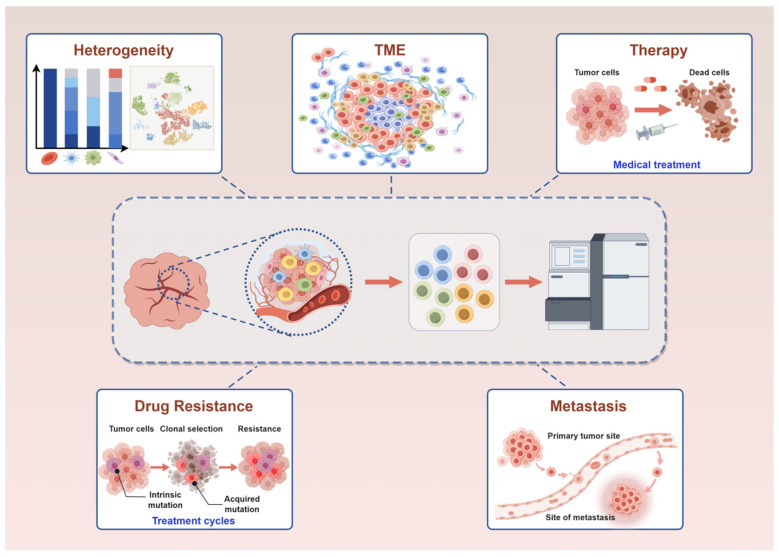
Application of scRNA-seq in the heterogeneity, tumor microenvironment, therapy, drug resistance and metastasis of breast cancer.

Breast cancer exhibits high heterogeneity, which is closely linked to the TME. The high heterogeneity and complex TME pose challenges for treatment, such as chemotherapy resistance. ScRNA-seq enables in-depth study of the heterogeneity and TME of breast cancer, facilitating the identification of relevant genes for precise treatment of breast cancer and providing new directions for the precision treatment, as well as insights into the mechanisms underlying chemotherapy resistance or metastasis during the treatment process (Figure 3). 

### 3.1. Application of scRNA-Seq in Exploring the Heterogeneity of Breast Cancer

The emergence of scRNA-seq has provided researchers with a powerful tool to uncover the immense complexity within tumors. The intrinsic nature of cancer involves heterogeneity, wherein cell populations within isogenic tumors exhibit varied cellular programs that collectively contribute to malignancy and diminish the efficacy of treatment [66]. Gray et al. [67] published a detailed classification of breast cells using a combination of scRNA-seq and complementary technologies. They created a comprehensive map of breast tissue and organoids in multiple dimensions. Similarly, Kumar et al. [68] developed the extensive human breast cell atlas with a focus on single-cell and spatial resolution. Through their analysis of single-cell transcriptomics, they examined 714,331 individual cells as well as 117,346 nuclei, uncovering 12 main cell categories and 58 distinct biological cell conditions. The results highlighted a rich population of immune cells and a broad spectrum of diverse breast epithelial cell states. Both studies demonstrate the diversity within breast tissue and offer a baseline for studying breast cancer in normal adult breast tissue.

In addition, heterogeneity is more pronounced in breast cancer. Liu et al. [69] performed scRNA-seq on primary breast tumors and concurrently analyzed fresh tumor samples using spatial transcriptomics. By examining the transcriptomes, they detected the presence of nine distinct cell clusters within the tumor based on canonical lineage markers. They further subdivided the epithelial cells, resulting in the identification of six subclusters. The copy number variation profiles of the tumor cells exhibited significant heterogeneity, indicating variability in the origin of these subclusters. Wu et al. [70] developed a single-cell intrinsic subtype classification (SCSubtype) approach to uncover the heterogeneity of breast cancer. By leveraging the characteristics of single cells, they further stratified the cohorts containing ER+, HER2+, and TNBC patients into nine subgroups, called “ecotypes”, which had unique cell composition and clinical results. This study provided a comprehensive transcriptional map of breast cancer and a more refined classification of breast cancer. Among different molecular subtypes of breast cancer, TNBC is characterized by high intra-tumoral variability. Liu et al. [71] identified four TNBC subtypes (Imm-E, Str-E, DR-E, and Met-E) in three bulk transcriptome datasets and a scRNA-seq dataset. Each subtype exhibited differences in immune signatures, stromal signatures, intratumor heterogeneity (ITH), signaling pathways, genomic stability, and prognosis. For instance, Imm-E exhibited the strongest immune signatures, had the lowest ITH, and boasted the best prognosis. In contrast, Met-E showed the highest ITH and had the worst prognosis. HER2-low status can be observed in certain patients diagnosed with TNBC, and this heterogeneity of HER2 is a vital factor in the management of TNBC. Hu et al. [72] conducted scRNA-seq on four HER2-negative and three HER2-low TNBC samples. They found that HER2-low TNBC exhibited more active metabolism and aggressive features, while HER2-negative TNBC exhibited a more active immune microenvironment, which was beneficial for achieving immune therapy responses. In summary, by establishing the single-cell atlas of breast cancer, these studies have further refined the classification of different types of breast cancer, uncovered the characteristics of different cell clusters and subclusters as well as the differences among different subtypes, and provided new insights for the refined classification of breast cancer and tailored treatment approaches.

Notably, the heterogeneity between breast cancer malignant cells and reference normal epithelial cells helps to reveal the origin of tumor cells. Hou et al. [73] integrated cells from normal breast tissues and breast cancer samples to construct a single-cell atlas. They investigated the heterogeneous origin of malignant cells and uncovered correlations between tumor stratification and clinical outcomes. Additionally, they determined the significant clinical implications of the luminal progenitor (LP) subtype in terms of prognosis and response to neoadjuvant chemotherapy, PARP inhibitors, and immunotherapy. This study revealed the evolution mimicry in the specification of breast cancer subtypes, providing a foundation for accurate prognostic and therapeutic stratification of breast cancer.

Moreover, there is an interaction among different cells in breast cancer, and this interaction is also accountable for the high heterogeneity. For example, Muciño-Olmos et al. [66] investigated the functional relationship among different cell subtypes in breast cancer cell lines and how this interdependence contributes to tumor development. They utilized scRNA-seq to analyze MCF7 multicellular tumor spheroids at two different time points during spheroid growth. The researchers identified three major cellular clusters that had distinct and complementary roles. While one cluster promoted proliferation, the others were involved in tissue invasion and acted as a reservoir population over time. These findings highlight the systemic nature of cancer, where cell populations exhibit task stratification to maintain tumor hallmarks.

In addition to heterogeneity among cells, there is also heterogeneity in gene expression within each tumor. Karaayvaz et al. [74] utilized scRNA-seq to analyze six primary tumors of TNBC and found significant variation in gene expression programs within each tumor, and much of this variation was influenced by clonal genomic copy number alterations. This suggests that the genetic composition of subpopulations drives their unique gene expression patterns. Through clustering gene expression profiles, researchers identified specific sets of cancerous cells present across multiple tumors. One subgroup, in particular, showed signs of resistance to treatment and metastasis, marked by heightened activity in glycosphingolipid metabolism and innate immune pathways. Their findings provide valuable insights into the intercellular heterogeneity and functional characteristics of TNBC tumors. In addition, the researchers identified five unique clusters of epithelial cells through clustering analysis. One of these clusters showed the highest percentage of actively dividing cells, suggesting increased proliferation capability. Upon further examination, it was discovered that this particular cluster exhibited characteristics resembling a luminal progenitor signature, which is regarded as the cell type accountable for the development of breast cancers [75]. Overall, this result highlights the connection between the functional heterogeneity of TNBC and genomic evolution and reveals the biological principles that lead to the poor prognosis of TNBC.

### 3.2. Application of scRNA-Seq in TME of Breast Cancer

The complex TME is one of the most significant factors contributing to the high heterogeneity of breast cancer, and the tumor immune microenvironment is of paramount importance. The most widely studied cells in the tumor immune microenvironment are T cells, B cells, and macrophages. Chung et al. [76] analyzed samples from 11 breast cancer patients using scRNA-seq and identified copy number variations that distinguish cancer cells from normal cells. At the individual cell level, carcinoma cells exhibited common characteristics within the tumor, while displaying significant heterogeneity in terms of breast cancer classifications and essential oncogenic pathways. The non-tumor cells were primarily immune cells, including T lymphocytes with regulatory or exhausted phenotypes, B lymphocytes, and macrophages with an M2 phenotype. Both T lymphocytes and macrophages exhibited immunosuppressive characteristics. These findings highlight the extensive intratumoral heterogeneity in the breast cancer transcriptome, which is affected by interactions between tumor cells and immune cells in the microenvironment surrounding the tumor. Azizi et al. [77] conducted scRNA-seq on 45,000 immune cells derived from eight breast carcinomas, as well as corresponding normal breast tissue, blood, and lymph node samples. Although they found significant similarities between immune cells in normal and tumor tissues, they observed ongoing phenotypic alterations specific to the tumor microenvironment. By analyzing paired scRNA-seq and T cell receptor sequencing data from an additional 27,000 T cells, they identified the combined influence of T cell receptor impact on variability in phenotype. These findings suggest a pattern of persistent stimulation in T cells, providing important insights for understanding tumor-infiltrating immune cells. Savas et al. [78] conducted scRNA-seq on 6311 T cells obtained from human breast cancers and observed notable diversity among the T cell population. They discovered that tumor cells with a substantial amount of tumor-infiltrating lymphocytes (TILs) contained CD8+ T cells exhibiting characteristics of tissue-resident memory T (TRM) cell differentiation. Based on the scRNA-seq data, the researchers created a gene signature unique to CD8+ TRM cells. This signature was strongly linked to enhanced survival outcomes in early-stage TNBC patients, offering more precise prognostic value compared to CD8 expression alone. The findings suggest that CD8+ TRM cells play a role in immune surveillance against breast cancer and are crucial targets for modulation through immune checkpoint inhibition. In another study [79], researchers conducted scRNA-seq on 117,958 immune cells and identified 31 distinct immune clusters. In TNBC, they observed a notable immunosuppressive environment characterized by abundant regulatory T cells (Tregs), reduced CD8+ T cells, and increased plasma cells. Tregs and fatigued CD8+ T cells in TNBC showed heightened immunosuppressive traits and dysfunction scores. Further analysis indicated a propensity for B cells in TNBC to undergo differentiation into plasma cells, driven by specific interactions between T cells and B cells. These findings contribute to a prognostic framework that effectively predicts outcomes for TNBC patients. In addition to T cells, B cells, and macrophages, natural killer (NK) cells also play a crucial role in tumor immunosurveillance [80]. Mao et al. [81] identified 44 significantly expressed NK cell-related genes (NKRG) via single-cell transcriptomics. Subsequently, they integrated machine learning to establish a breast cancer prognostic signature based on these NKRGs. For breast cancer patients in the high-risk group, the TME was likely to be more disposed to inhibiting the immune response, thus facilitating the survival and proliferation of tumor cells. The above studies have analyzed the dynamic changes of immune cells in the TME, and revealed the heterogeneity of the breast cancer immune microenvironment and the interaction mechanism between tumor cells and immune cells. They have emphasized the significance of immune cells in regulating the tumor immune microenvironment, immune surveillance, immunotherapy, and clinical prognosis evaluation.

In the tumor interstitial microenvironment, cancer-associated fibroblasts (CAFs) play a key role in remodeling the TME, and they represent a highly prevalent and abundant cellular population within the TME [82,83]. Ma et al. [83] identified four subgroups of CAFs with different functions and described their spatial distribution through a combined examination of spatial and single-cell transcriptomics for six prevalent cancer types, including breast cancer. Moreover, another study broke down the cell types even further. In the study of Cords et al. [84], they analyzed a scRNA-seq dataset obtained from 14 human breast cancer samples, encompassing approximately 119,000 cells. Through unsupervised clustering, this research identified 16,704 stromal cells, with nine distinct populations classified as CAFs and one population as pericytes. Based on RGS5 expression, 2389 stromal cells were identified as pericytes, and a total of 14,315 CAFs were identified. They utilized this dataset to investigate the diverse phenotypes of fibroblasts within breast tumors. This classification of CAFs provides a framework for comparing CAF characteristics across studies, facilitates analysis of their functional responsibilities, and may potentially inform the development of new treatment strategies in the future. Recently, Ning et al. [85] mapped the landscape of CAFs and characterized the heterogeneity of fibroblasts in breast cancer. Significantly, they identified a new subtype, SFRP4+ CAF, which inhibited migration and predicted the prognosis of breast cancer. This result is anticipated to serve as a potential biomarker for precise prognostic evaluation and therapeutic intervention in breast cancer. Most notably, the relationship between CAFs and the immune microenvironment has recently received much attention. Croizer et al. [86] deciphered the plasticity of inflammatory and myofibroblastic CAF (iCAF/myCAF) in breast cancer. They identified the transitions of detoxification-associated iCAF (Detox-iCAF) into the immunosuppressive extracellular matrix (ECM)-producing myCAF (ECM-myCAF), which were dependent on DPP4 and YAP-1. In turn, ECM-myCAF cells contribute to creating immunosuppressive environments by polarizing regulatory NK cells, TREM2+ macrophages, and T cells. Another study explored the origins of iCAF and myCAF, providing ideas for developing targeted therapies based on CAF [87]. Through scRNA-seq and a series of validation experiments, they confirmed that CAFs originated from mammary tissue-resident normal fibroblasts (NFs). Specifically, the CD26+ NF population ultimately transformed into iCAF, and the CD26- NF population ultimately transformed into myCAF [87]. Together, scRNA-seq is mainly used in the interstitial microenvironment of breast cancer to analyze the subtypes, functions, origins of CAFs, and their relationship with the immune microenvironment. Its clinical significance lies in providing potential biomarkers for accurate prognostic assessment and therapeutic intervention of breast cancer and also providing ideas and frameworks for the development of new treatment strategies.

### 3.3. Application of scRNA-Seq in Therapy of Breast Cancer

The complex TME caused by the heterogeneity of breast cancer has brought challenges to the treatment of breast cancer. scRNA-seq technology can be employed to open up a way for precision treatment of breast cancer. Gambardella et al. [88] conducted transcriptional profiling on 35,276 individual cells isolated from 32 breast cancer cell lines, creating a comprehensive single-cell atlas. Moreover, they integrated data from extensive in vitro drug screening with the single-cell atlas to predict drug responses using computational methods based on single-cell profiles. Notably, their findings indicated that transcriptional heterogeneity facilitates cells with varying drug sensitivity to coexist within the same population. This knowledge of drug sensitivity in cancer cell lines can potentially guide personalized drug treatment for patients, especially when bulk gene expression profiles are available.

Neoadjuvant therapy (NAT) has emerged as a crucial component of comprehensive breast cancer treatment. Nevertheless, owing to the heterogeneity of breast cancer, substantial variations in NAT outcomes persist. ScRNA-seq analysis holds great potential in this regard as it can facilitate the screening of biomarkers, thereby enabling the identification of subgroups of breast cancer patients who are likely to benefit from NAT. In a study conducted by Huang et al. [89], 16 genes associated with residual cancer burden (RCB) after NAT and affecting patients’ distant recurrence-free survival (DRFS) were identified, and GATA3 was ultimately selected as a key gene signature with a high RCB index. At the single-cell level, GATA3 was mainly enriched in mast cells, and high GATA3 expression showed more mast cell infiltration. Moreover, patients with high expression of GATA3, elevated levels of resting mast cell infiltration, and a large proportion of ER-positive were conducive to attaining better DRFS. Zhang et al. [90] integrated scRNA-seq and bulk RNA-seq analysis and identified 190 characteristic genes associated with the prognostic response to NAT. They further established a prognostic risk model that is specifically associated with NAT and evaluated the potential influence of risk scores on drug response. In addition, Mei et al. [91] classified breast cancer patients into the tumor-rich subtype and the immune-infiltrating subtype through scRNA-seq. Moreover, they identified CRABP2 and CD69 as biomarkers for the tumor enrichment type and immune invasion type, respectively. They also demonstrated that the subtypes with high expression of CRABP2 and low expression of CD69 had the worst prognosis and the lowest sensitivity to NAT.

Recently, chemotherapy combined with immunotherapy has been explored due to the need for more effective treatment strategies considering the complex tumor microenvironment and the limitations of single-modality therapies. The most challenging treatment is for TNBC, as there is currently no corresponding target available for targeted therapy. In the study conducted by Deng et al. [92], scRNA-seq was utilized to investigate the alterations in immune cells within tumor biopsies from two patients with advanced TNBC who underwent disparate treatment regimens. scRNA-seq analysis uncovered notable diversity within tumor-infiltrating immune cell populations. Additionally, the study discovered that chemotherapy and immunotherapy influenced the tumor microenvironment. In the patient who responded favorably to treatment, there was a decrease in PD-1 high-expressing T cells and the presence of tissue-resident memory T cells (TRM) after receiving nab-paclitaxel plus pembrolizumab. This reveals that in TNBC patients, different treatment regimens targeting the TME may lead to significant heterogeneity in the tumor-infiltrating immune cell community. Such findings help us to gain insight into the influence of different treatment strategies on tumor immune response and are expected to provide important clues for individualized treatment. In addition, by using scRNA-seq and ATAC sequencing, Zhang et al. [93] examined immune cell dynamics in 22 patients with advanced TNBC who were undergoing treatment with paclitaxel or a combination of paclitaxel and the anti-PD-L1 drug atezolizumab. The study revealed that elevated baseline levels of CXCL13+ T cells are associated with traits of proinflammatory macrophages. Following combination therapy, CXCL13+ T cells and classical type 1 dendritic cells significantly increased, whereas they decreased after paclitaxel treatment alone. These findings emphasize the critical role of CXCL13+ T cells in efficient reactions to anti-PD-L1 therapy and highlight concerns regarding the potential implications of reducing these cells when combining paclitaxel with atezolizumab for treating TNBC. Both studies have conducted in-depth analyses of the changes in the immune microenvironment and immune cell dynamics of breast cancer resulting from chemotherapy combined with immunotherapy. They have highlighted the role and concerns of specific immune cells in combined therapy, which could potentially provide important clues for individualized treatment.

### 3.4. Application of scRNA-Seq in Drug Resistance of Breast Cancer

The treatment of metastatic breast cancer remains a significant challenge, as a portion of patients eventually experience chemotherapy resistance. The development of acquired drug resistance is primarily attributed to subclones within heterogeneous tumors that are resistant to treatment [94]. Although clinical treatment regimens have demonstrated efficacy in a substantial number of breast cancer patients, a subset of individuals experienced limited response to treatment due to the development of drug resistance, ultimately leading to reduced overall survival. Currently, the precise biochemical pathways responsible for the development of drug resistance in breast cancer remain unclear. Scientists are still debating whether drug resistance arises from pre-existing genes in the body or from newly acquired genetic mutations. ScRNA-seq can help resolve this dilemma [95].

TNBC is a subtype that often develops resistance to chemotherapy, posing a significant challenge to its treatment. To overcome this challenge, Kim et al. [96] employed scRNA-seq to analyze the response of tumor cells in 20 patients with TNBC to neoadjuvant chemotherapy (NAC). They found that NAC led to either clonal extinction or persistence in different patients. Detailed analysis of eight patients revealed that resistant genotypes were already present and selectively favored by NAC, while chemotherapy induced changes in transcriptional profiles through reprogramming. The clonal extinction group showed complete elimination of tumor cells, leaving normal cell types behind, while the clonal persistence group had residual tumor cells with altered genotypes and phenotypes. Their findings show that certain genotypes chosen by NAC had already expressed chemoresistant genes before treatment and were ready for transcriptional reprogramming. They also observed that in chemoresistant tumor cells post-NAC, some gene signatures, such as CDH targets, epithelial-mesenchymal transition (EMT), angiogenesis, ECM degradation, hypoxia, and the AKT1 signaling pathway via mTOR, were upregulated. This finding is crucial in assessing a patient’s potential response to chemotherapy before NAC, aiding in the clinical customization of NAC regimens. Overall, their data support a model of chemoresistance involving both pre-existing adaptive genotypes and acquired changes in tumor cells. In addition, Shaath et al. [97] used scRNA-seq to identify long non-coding RNA (lncRNA) transcriptional profiles associated with resistance to NAC in TNBC. A comparison of lncRNA transcriptomes in single cells during NAC treatment revealed minimal overlap based on lncRNA transcriptomes, indicating a significant effect of NAC on lncRNA transcription. Differential analysis revealed abnormally expressed lncRNA transcripts, highlighting the crucial role of MALAT1 lncRNA in TNBC pathogenesis and resistance to NAC. Further validation experiments showed that MALAT1 promoter deletion enhanced the sensitivity of TNBC to paclitaxel and doxorubicin, and changed the expression pattern of other lncRNAs, which confirmed the important role of MALAT1 in driving NAC resistance in TNBC. These findings are crucial for evaluating TNBC patients’ potential response to NAC and customizing NAC regimens while providing insights into the mechanisms of chemotherapy resistance and potential targets for overcoming resistance.

In addition, other researchers utilized scRNA-seq to conduct comprehensive analyses of the MCF7 luminal breast cancer cell line and its docetaxel-resistant derivatives [98]. They observed an upregulation of genes linked to epithelial-to-mesenchymal transition and stemness, as well as a downregulation of cell-cycle-related genes in the drug-resistant cells, primarily regulated by LEF1. Noteworthy, a few cells in the original cell population showed a gene expression pattern similar to drug-resistant cells, indicating a rare subset of stem-like cells with a natural tendency towards docetaxel resistance. This study emphasizes the impact of intratumor heterogeneity in breast cancer on drug resistance at the single-cell level [98]. Tamoxifen is also a commonly used endocrine therapy drug for luminal breast cancer. By means of scRNA-seq, Gao et al. [99] identified a novel subset of CD63+ CAFs that can induce tamoxifen resistance in ER+ breast cancer. This subset of cells is capable of secreting exosomes rich in miR-22, which can bind to the target ERα and the main negative regulator PTEN of the PI3K-AKT pathway, resulting in a reduction in the expression of ERα and PTEN. Thus, it endows cancer cells with tamoxifen resistance, suggesting that CD63+CAFs may be a new therapeutic target to enhance tamoxifen sensitivity.

There are also studies of drug resistance in HER2-positive breast cancer. Trastuzumab is widely employed to treat HER2-positive breast cancer; however, it also encounters resistance challenges. By utilizing scRNA-seq, Du et al. [100] identified a novel subpopulation of podoplanin-positive (PDPN+) CAFs. In HER2+ breast cancer, trastuzumab resistance is induced by inhibiting NK cell-mediated antibody-dependent cell-mediated cytotoxicity (ADCC). Additionally, they found that PDPN+CAFs enriched in trastuzumab-resistant tumor tissues and promoted trastuzumab resistance in HER2+ breast cancer by secreting immunosuppressive factors, thereby inhibiting ADCC mediated by functional NK cells. Moreover, in the trastuzumab-resistant cell lines of SK-BR-3 and BT-474, scRNA-seq assay revealed single nucleotide variations (SNVs) related to trastuzumab resistance [101]. These include AIFM1 P548L and IL1RAPL2 S546C in SK-BR-3 cells, as well as ANAPC4 E16K and MFSD11 L242I in BT-474 cells, which could lead to a decrease in the sensitivity to trastuzumab [101]. This study speculated that the concurrent SNVs related to trastuzumab resistance might be a potential target for the treatment of patients with trastuzumab resistance.

It is worth noting that some non-inflammatory tumors, including immune-desert and immune-excluded tumors, often have resistance to immunotherapy. Yi et al. [102] combined YM101 (anti-TGF-β/PD-L1 bispecific antibody) with MSA-2 (oral STING agonist) for the treatment of non-inflammatory tumors. scRNA-seq analysis indicated that MSA-2 works synergistically with YM101 to enhance the immune response against tumors, and effectively overcome resistance to immunotherapy in an orthotopic mouse breast cancer model characterized by immune exclusion and immune desertion, providing a new treatment strategy for such tumors.

### 3.5. Application of scRNA-Seq in Metastasis of Breast Cancer

The metastasis of breast cancer is a primary challenge in clinical treatment, referring to the movement of neoplastic cells from the primary tumor to metastatic sites, and is a leading cause of mortality in breast cancer patients. The metastasis of breast cancer is a complicated and multistage process, including the infiltration of cancer cells from the primary site into surrounding tissues, then entering blood vessels or lymphatic channels to survive in the body, and finally extravasating to other organs from the blood vessels, forming tumors in different locations.

Metastasis to the axillary lymph nodes is the most prevalent form. Nevertheless, the detailed mechanisms of breast cancer metastasis are still largely unknown. Xu et al. [103] performed an investigation of single-cell transcriptome data from primary breast tumors and metastatic axillary lymph nodes, identifying nine distinct cell subpopulations, including different types of breast cancer stem cells (BCSCs). These BCSCs exhibited a copy-number variation pattern similar to that of normal breast tissue and demonstrated the ability to differentiate into multiple cell types. By examining the genomes of individual cells, they identified specific mutations connected to lymphatic spread in breast cancer. Their findings confirmed that BCSCs originate from normal breast tissue and are predominantly located in primary breast tumors. Additionally, BCSCs have the potential to develop into more metastatic cancer cell clusters, facilitating their spread to lymph nodes. These findings highlight the significance of BCSCs in breast cancer progression and provide valuable insights into the mechanisms of lymph node metastasis in breast cancer. In addition, other researchers performed scRNA-seq on primary tumors, negative lymph nodes (NLs), and positive lymph nodes (PLs) from breast cancer patients. They identified a previously unrecognized cell subpopulation within the PL of breast cancer patients, characterized by abnormally high expression of CXCL14. Cell trajectory analysis further indicated that CXCL14 expression increased during the late pseudo-time. Their research outcomes have the potential to advance the current comprehension of the mechanisms underlying lymph node metastasis [104]. Axillary lymph node metastasis has also been studied by other scholars. In a study by Liu et al. [105], scRNA-seq was employed to analyze four pairs of primary invasive breast tumors and metastatic axillary lymph nodes. The research revealed that early disseminated breast cancer cells transition from glycolysis to oxidative phosphorylation during the process of metastasis. Notably, the heightened oxidative phosphorylation signifies a temporary phase in the dissemination process, as the colonized disseminated cells subsequently demonstrate more aggressive characteristics with increased glycolysis. This investigation provides insight into the metabolic changes within disseminated cells and offers a valuable understanding of the dynamic progression of breast cancer cells during the initial phases of dissemination. In addition, podoplanin-expressing macrophages have been shown to be associated with lymphatic metastasis of breast cancer [106]. Shen et al. [107] conducted a transcriptomic analysis of individual cells from breast cancer patients, including primary tumors, NLs, and PLs, using publicly available datasets. They found an accumulation of M2 macrophages in NLs and PLs. Furthermore, the activation and migration ability of M2 macrophages from primary tumors were significantly higher compared to non-lymph nodes and PLs. Several genes related to M2 macrophage activation and migration were upregulated in primary tumors. The proportion of activated and migrating M2 macrophages, along with the GSVA enrichment score, could potentially serve as markers for lymph node metastasis in breast cancer. In summary, Shen et al. identified M2 macrophages and their activation and migration-related genes as potential indicators of lymph node metastasis in breast cancer. Recently, Sanjaya et al. [108] employed scRNA-seq to compare the genetic signatures between lymph node metastases and primary breast tumors. Differential gene analysis revealed that there were 43 upregulated genes and 4 downregulated genes in primary tumors. They also discovered that in the initial phase of lymph node metastasis, the loss of differentiation and tumor suppressor pathway activity, as well as the interaction between cells and ECM, all play a role in promoting metastasis. In addition, several genes associated with EMT were abundantly expressed in metastatic lesions, further strengthening the important role of EMT in cancer metastasis.

In addition to female breast cancer (FBC), males can also develop breast cancer. Researchers have studied male breast cancer (MBC), a rare malignant tumor. Sun et al. [109] conducted a comparison of scRNA-seq data from MBC and FBC samples to assess their scores for signatures related to metastasis. The findings indicated that MBC cells exhibited elevated scores for metastatic signatures compared to FBC cells, especially in terms of angiogenesis and cellular migration. These findings suggest that MBC has a greater potential for metastasis. In summary, Sun et al. identified higher metastasis-related signature scores in MBC, indicating its increased metastatic potential compared to FBC.

In conclusion, scRNA-seq is widely used in breast cancer. These applications are summarized in Table 2.

## 4. Potential Future Directions of scRNA-Seq in Breast Cancer Research

In breast cancer research, scRNA-seq has emerged as a powerful tool for understanding the complex biology of the disease. Looking ahead, there are several potential future directions for scRNA-seq that could further enhance our knowledge and lead to improved treatment strategies.

One promising avenue is the integration of scRNA-seq with other omics data, such as proteomics, spatial transcriptomics, and epigenomics, which can offer a broader perspective on the molecular landscape of breast cancer. On the one hand, the integration of multiple omics analysis can help researchers with unicellular sequencing of cross-validation data with other omics data, so as to increase the reliability of the data and the accuracy. On the other hand, multi-omics analysis can also reveal the interrelationships and regulatory networks between different omics levels, providing deeper insights into cell biology and disease research. For example, integrating scRNA seq with spatial transcriptomics can reveal the spatial heterogeneity of cells within the tumor microenvironment, shedding light on how different cell types interact and contribute to tumor progression and metastasis [110]. By integrating scRNA-seq with DNA methylation data, researchers can identify genes that are silenced or activated by methylation changes and understand how these epigenetic modifications contribute to tumorigenesis [111]. In summary, this multi-dimensional approach can help identify novel therapeutic targets, predict treatment responses, and develop personalized treatment strategies.

Improvements in computational tools are another area of potential progress. Analysis of scRNA-seq data routinely involves machine learning methods, such as feature learning, clustering, and classification, to assist in uncovering novel information from scRNA-seq data. However, current methods are not well suited to deal with the substantial amount of noise that is created by the experiments or the variation that occurs due to differences in the cells of the same type. To address this, Srinivasan et al. [112] developed a new hybrid approach, deep unsupervised single-cell clustering (DUSC), which integrates feature generation based on a deep learning architecture by using a new technique to estimate the number of latent features, with a model-based clustering algorithm, to find a compact and informative representation of the single-cell transcriptomic data generating robust clusters. They also include a technique to estimate an efficient number of latent features in the deep learning model. Their method outperforms both classical and state-of-the-art feature learning and clustering methods, approaching the accuracy of supervised learning. They applied DUSC to a single-cell transcriptomics data set obtained from a TNBC tumor to identify potential cancer subclones accentuated by copy-number variation and investigate the role of clonal heterogeneity. In addition to that, reconstructing gene regulatory networks (GRNs) from scRNA-seq data reveals insights into cell differentiation but is challenging due to gene expression variability. To address this, Wang et al. [113] developed DeepRIG, which uses a graph autoencoder (GAE) model to reconstruct GRNs from single-cell data. DeepRIG calculates gene correlation coefficients and creates a robust prior regulatory map based on co-expression patterns to manage noise. By converting GRNs into gene maps it simplifies complex regulatory relationships using neighborhood information. DeepRIG’s GAE-based approach integrates global regulatory information with gene expression profiles to accurately infer GRNs in a semi-supervised manner. Its effectiveness was validated on synthetic and real scRNA-seq datasets. Applied to TNBC samples, DeepRIG identified ten genes with significant topological features, including four known to be involved in TNBC, demonstrating its potential for discovering new regulators or targets in cancer research. These techniques all belong to the refinement of computational tools for the analysis of traditional scRNA-seq data. With the help of these technologies, the biological information revealed by scRNA-seq can be more deeply analyzed. They add new power to breast cancer research and are potential future directions of scRNA-seq in breast cancer research.

## 5. Conclusions

Traditionally, researchers obtain data such as gene expression, protein expression, and metabolic information of cell populations by population averaging. However, this approach masks heterogeneity and individual differences between different cells. The emergence of scRNA-seq technology has changed this situation, which can obtain genome, transcriptome and epigenome data of single cells at high throughput. In this review, the application of scRNA-seq in breast cancer has made significant advancements in cell heterogeneity, tumor microenvironment, therapy, drug resistance, and metastasis (Figure 3, Table 2). It has enabled researchers to enhance their comprehension of breast cancer by uncovering unprecedented insights into the cellular heterogeneity and complexity of breast cancer. This cutting-edge technology facilitates the profiling of gene expression at the single-cell level, uncovering diverse cell types and states within the tumor microenvironment. By dissecting the cellular composition and transcriptional signatures of breast cancer cells, scRNA-seq provides new perspectives for enhancing insight into mechanisms involved in cancer treatment, chemotherapy resistance, and metastasis in breast cancer. With the continuous development and maturity of single-cell technology and other omics technologies, multi-omics analysis will become an important tool for future life science research, helping to deepen our understanding of breast cancer.

## Figures and Tables

**Table 1:** Technical characteristics of single-cell transcriptomic sequencing technologies.

Method	RNA-Capture	Transcript Coverage	UMI	Amplification Technology
Tang	polyA	full length	No	PCR after polyA tailing
Quartz-seq	polyA	full length	No	
Quartz-seq2	polyA	full length	Yes	
SUPeR-seq	polyA	full length	No	
MATQ-seq	polyA	full length	Yes	
SMART-seq	polyA	full length	No	Template-switching-based PCR
SMART-seq2	polyA	full length	No	
SMART-seq3	polyA	full length	Yes	
FLASH-seq	polyA	full length	Yes	
STRT-seq	polyA	5′ tag	Yes	
STRT-seq-2i	polyA	5′ tag	Yes	
SCRB-seq	polyA	3′ tag	Yes	
Drop-seq	polyA	3′ tag	Yes	
CEL-seq	polyA	3′ tag	Yes	In vitro transcription(IVT)
CEL-seq2	polyA	3′ tag	Yes	
MARS-seq	polyA	3′ tag	Yes	
MARS-seq2.0	polyA	3′ tag	Yes	
In Drops	polyA	3′ tag	Yes	

Abbreviations: scRNA-seq, single-cell RNA-sequencing; UMI, unique molecular identifier; PCR, polymerase chain reaction.

**Table 2:** Various applications of scRNA-seq in breast cancer.

Applications	Category	Study	Clinical Significance	References
Heterogeneity	Heterogeneity within normal breast tissues	Breast cell typing. Development of the human breast cell atlas.	Demonstrating the diversity within breast tissue. Offering a baseline for studying breast cancer in normal adult breast tissue.	[67,68]
	Heterogeneity within breast tumors	Development of the breast cancer cell atlas. More refined classification of different types of breast cancer. Revealing the characteristics of different cell clusters and subclusters, as well as the differences of different subtypes.	Offering insights into the refined classification and tailored therapies for breast cancer.	[69,70,71,72]
	Heterogeneity between breast cancer malignant cells and reference normal epithelial cells	Revealing evolution mimicry during the specification of breast cancer subtype.	Revealing the origin of tumor cells and providing a foundation for accurate prognostic and therapeutic stratification of breast cancer.	[73]
	Heterogeneity among breast cancer cell lines	Investigation of the functional relationship among different cell subtypes in breast cancer cell lines and how this interdependence contributes to tumor development.	Highlighting the systemic nature of cancer and task stratification of cell populations to maintain tumor hallmarks.	[66]
	Heterogeneity in gene expression within each tumor	Revealing the phenotypes and biology underlying the genetic evolution and clinical behavior of TNBC.	Highlighting the connection between the functional heterogeneity of TNBC and genomic evolution, and revealing the biological principles that lead to the poor prognosis of TNBC.	[74]
TME	Tumor immune microenvironment(T cells, B cells, macrophages, NK cells)	Revealing the heterogeneity characteristics of the immune microenvironment. Analyzing the dynamic changes of immune cells in the tumor microenvironment. Revealing the interaction between tumor cells and immune cells.	Providing more accurate prognostic value. Revealing the role of immune cells in immune surveillance. Providing targets for immunotherapy.	[76,77,78,79,80,81]
	Tumor interstitial microenvironment (CAFs)	Identifying different subgroups of CAFs with distinct functions. Understanding the plasticity of CAFs and their relationship with the immune microenvironment. Investigating the origins of CAFs.	Providing potential biomarkers for accurate prognostic. Providing ideas and frameworks for the development of new treatment strategies.	[83,84,85,86,87]
Therapy	Drug sensitivity	Predicting drug sensitivity	Guiding personalized drug treatment for patients.	[88]
	Predictive markers for NAT	Screening for biomarkers associated with the prognostic response to NAT.	Enabling the identification of subgroups of breast cancer patients who are likely to benefit from NAT.	[89,90,91]
	Chemotherapy combined with immunotherapy	Analyses on the changes in the immune microenvironment and immune cell dynamics of breast cancer resulting from chemotherapy combined with immunotherapy.	Highlighting the role and concerns of specific immune cells in combined therapy, which could potentially provide important clues for individualized treatment.	[92,93]
Drug resistance	Drug resistance of TNBC	Exploration of drug resistance mechanisms. Developing potential targets to overcome drug resistance.	Customizing treatment regimens. Providing insights into the mechanisms of chemotherapy resistance. Providing potential targets for overcoming resistance.	[96,97]
	Drug resistance of luminal breast cancer	Identification of subpopulations of drug-resistant cells. Exploration of drug resistance mechanisms.	Providing insights into the mechanisms of chemotherapy resistance. Providing potential targets for overcoming resistance.	[98,99]
	Drug resistance of HER2-positive breast cancer	Identification of drug resistance-related cell subpopulations and single nucleotide variations. Exploration of drug resistance mechanisms.	Providing insights into the mechanisms of chemotherapy resistance. Providing potential targets for overcoming resistance.	[100,101]
	Drug resistance of non-inflammatory breast cancer	The role of combined application of MSA-2 and YM101 in immune therapy resistance of non-inflammatory tumors.	Providing a new treatment strategy for non-inflammatory tumors.	[102]
Metastasis	Lymph node metastasis in female breast cancer	Identification of subpopulations related to lymph node metastasis. The mechanisms underlying lymph node metastasis. The metabolic changes during metastasis. Changes in immune microenvironment during metastasis. Genetic signatures between lymph node metastases and primary breast tumors.	Understanding the detailed mechanism of breast cancer metastasis. Providing new markers for lymph node metastasis of breast cancer and offering a reference for clinical diagnosis and treatment.	[103,104,105,106,107,108]
	Metastasis in male breast cancer	Metastatic characteristics of male breast cancer.	Providing a new perspective for the research and treatment of male breast cancer.	[109]

Abbreviations: TNBC, triple-negative breast cancer; TME, tumor microenvironment; NK, natural killer; CAFs, cancer-associated fibroblasts; NAT, neoadjuvant therapy; HER2, human epidermal growth factor receptor-2.
